# Joint Identification of Genetic Variants for Physical Activity in Korean Population

**DOI:** 10.3390/ijms150712407

**Published:** 2014-07-14

**Authors:** Jayoun Kim, Jaehee Kim, Haesook Min, Sohee Oh, Yeonjung Kim, Andy H. Lee, Taesung Park

**Affiliations:** 1 Biomedical Research Institute, Seoul National University Bundang Hospital, Seongnam-si 463707, Korea; E-Mail: nunadli@gmail.com; 2 Interdisciplinary Program in Bioinformatics, Seoul National University, Seoul 151742, Korea; E-Mail: wisewidewarm@gmail.com; 3 Division of Epidemiology and Health Index, Center for Genome Science, Korea Centers for Disease Control & Prevention, Cheongwon-gun 363951, Korea; E-Mails: agnesmin@korea.kr (H.M.); kimye@korea.kr (Y.K.); 4 Department of Biostatistics, SMG-SNU Boramae Medical Center, Seoul 156707, Korea; E-Mail: oh.sohee@gmail.com; 5 School of Public Health, Curtin University, Perth 6845, Australia; E-Mail: Andy.Lee@curtin.edu.au; 6 Department of Statistics, Seoul National University, Seoul 151742, Korea

**Keywords:** elastic-net regularization, empirical replication, metabolic equivalent task

## Abstract

There has been limited research on genome-wide association with physical activity (PA). This study ascertained genetic associations between PA and 344,893 single nucleotide polymorphism (SNP) markers in 8842 Korean samples. PA data were obtained from a validated questionnaire that included information on PA intensity and duration. Metabolic equivalent of tasks were calculated to estimate the total daily PA level for each individual. In addition to single- and multiple-SNP association tests, a pathway enrichment analysis was performed to identify the biological significance of SNP markers. Although no significant SNP was found at genome-wide significance level via single-SNP association tests, 59 genetic variants mapped to 76 genes were identified via a multiple SNP approach using a bootstrap selection stability measure. Pathway analysis for these 59 variants showed that maturity onset diabetes of the young (MODY) was enriched. Joint identification of SNPs could enable the identification of multiple SNPs with good predictive power for PA and a pathway enriched for PA.

## 1. Introduction

Physical activity (PA) is any bodily movement produced by skeletal muscles that requires energy expenditure [1]. It includes exercise as well as work and recreational activities which involve bodily movements. PA can be quantified by metabolic equivalent task (MET) intensity [1,2], and plays an important role in the morbidity, mortality, and health care costs of obesity and related chronic diseases [3].

The behavior of PA may be determined by genetic factors [4,5,6]. In particular, between 48% and 71% of the variability in adult exercise behavior can be explained by genetic factors [6]. Indeed, evidence from twin and family studies have suggested that genetic factors contribute to the propensity of being sedentary [5]. A novel approach has been proposed to identify genetic variants that are related to leisure-time exercise behavior, by conducting Genome-Wide Association (GWA) analyses using logistic regression to find genes associated with exercisers and non-exercisers [7]. Recently, a genome-wide study with quantitative PA as a phenotype was performed for the Korean population [8]. This study revealed how to define phenotypes of PA in genetic association studies, together with appropriate statistical methods for their analysis [8].

In reporting genetic variants associated with a trait or disease, most traditional GWA studies adopted a single-marker approach that identifies single genetic factors one by one. However, this method is inefficient in predicting joint effects of multiple genetic variants on the common complex trait [9,10]. A multiple-marker approach is the preferred alternative for their joint identification [11]. However, multiple linear and logistic regression models are often ill-defined in GWA studies when the number of predictor variables is larger than the sample size. In addition, collinearity often occurs between predictor variables due to linkage disequilibrium among single nucleotide polymorphisms (SNPs).

To identify multiple genetic variants for common complex traits or diseases, an elastic-net (EN) regularization method had been proposed by Cho *et al.* [11], along with some consistency measures based on bootstrap sampling. The EN regularization method was originally introduced for model fitting and variable selection in ill-defined multiple regressions. It has been applied to GWA studies [12,13] and provided a better prediction than those based on ordinary regression models [14,15] when variables are larger than sample sizes and multicollinearity problems may exist.

The present study aimed to find genetic variants influencing PA in the Korean population. Single-SNP association tests were initially conducted to assess genetic associations between daily PA and various SNP markers. We then performed multiple SNP analysis with EN regularization to determine genetic variants associated with PA. The SNPs identified provide novel biological evidence to understand the genetics of PA through pathway enrichment analysis.

## 2. Results and Discussion

### 2.1. Results

#### 2.1.1. Physical Activity Levels

**Figure 1:**
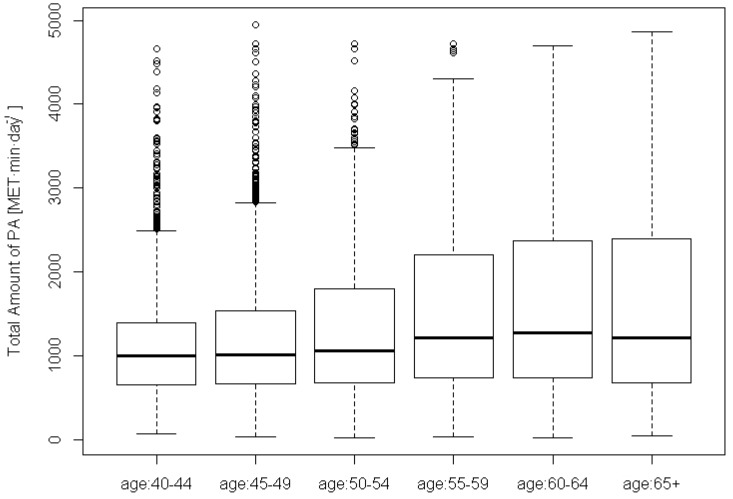
Box plots of total amount of physical activity (PA) by age group (40–44, 45–49, 50–54, 55–59, 60–64, 65+).

Overall, the average daily PA level was 1332 (SD 871) MET·min for the Korean participants. Men (mean 1367, SD 887 MET·min·day^−1^) appeared to be slightly more active than women (mean 1300, SD 856 MET·min·day^−1^). The mean PA level of the Ansung cohort (1678, SD 1046 MET·min·day^−1^) was higher than that of the Ansan cohort (1038, SD 534 MET·min·day^−1^), suggesting that people in the rural community tended to be more active than their city counterparts. Figure 1 shows the box plots of self-reported PA by age groups (40–44, 45–49, 50–54, 55–59, 60–64, 65+). Although the median PA levels of these six age groups were similar, the PA distributions exhibited substantial variations. In particular, the majority of younger participants appeared to sustain low PA levels with the exception of a few outliers. For older participants especially those over 65 years, their PA levels varied considerably between individuals, as evident from the wide interquartile ranges.

#### 2.1.2. Individual Single Nucleotide Polymorphism (SNP)-Based Association Analysis

**Figure 2:**
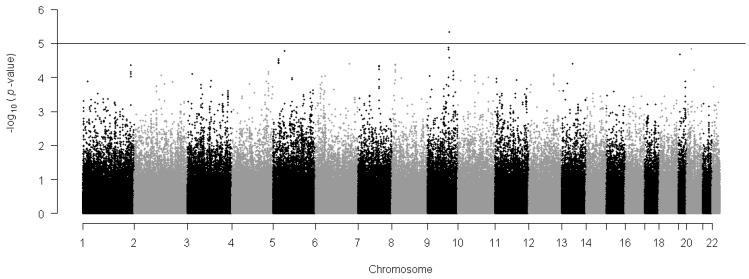
Manhattan plot showing total amount of PA. The horizontal reference line represents the genome-wide association (GWA) threshold *p*-values 10^−5^. The *p*-values from single SNP association test is indicated in −log_10_ scale against each chromosome.

Single-marker association analysis was performed for individual SNP with sex, age, area, and body mass index as covariates. Table 1 presents the results of the single-SNP association tests. The first six columns give the SNP information and the remaining columns summarize the regression results. Figure 2 further shows the Manhattan plot of 344,893 SNPs, where the *y*-axis represents the log-transformed *p*-value and the *x*-axis represents the chromosomes. The horizontal solid line indicates *p*-value = 10^−5^. Although these SNPs did not achieve the genome-wide level significance, 41 genetic variants (listed in Table 1) emerged among the 344,893 SNPs to have some evidence of association with PA under *p*-value < 10^−4^, and they were mapped to 27 genes.

**Table 1:** Single nucleotide polymorphisms (SNPs) associated with physical activity by single-SNP association tests (*p*-value < 10^−4^).

rs Number	Gene Symbol	Location of SNP	Cytoband	Minor Allele	MAF ^a^	BETA ^b^	*p*-Value ^c^
rs7023003	*RN7SK*, *SLC44A1*	intergenic	9q31.1d	G	0.2522	65.58	4.67 × 10^−^^5^
rs11791649		intergenic	9q31.1b	A	0.0681	107.6	1.30 × 10^−5^
rs6074898	*MACROD2*	intronic	20p12.1c	C	0.0598	113.9	1.42 × 10^−5^
rs17228531		intergenic	9q31.1b	A	0.0676	107.1	1.49 × 10^−5^
rs10057067	*ITGA1*	intronic	5q11.2b	G	0.4550	−53.42	1.67 × 10^−5^
rs12462609	*CACNA1A*	intronic	19p13.13b	A	0.1120	−83.29	2.04 × 10^−5^
rs7020422	*RN7SK, SLC44A1*	intergenic	9q31.1d	A	0.2350	61.84	2.59 × 10^−5^
rs11952141		intergenic	5p15.1a	C	0.1833	67.73	2.92 × 10^−5^
rs6867384		intergenic	5p15.1a	G	0.1838	67.28	3.18 × 10^−5^
rs6891956		intergenic	5p15.1a	T	0.1839	66.67	3.66 × 10^−5^
rs6880596		intergenic	5p15.1a	A	0.1767	67.66	3.78 × 10^−5^
rs17069951	*CITED2*	intergenic	6q24.1b	T	0.0106	246.8	3.91 × 10^−5^
rs10507652	*TDRD3*	intergenic	13q21.2b	T	0.0536	−113.1	3.95 × 10^−5^
rs11781985	*MFHAS1*, *CLDN23*	intergenic	8p23.1d	C	0.0632	105.8	4.23 × 10^−5^
rs940031	*CLDN23*	intergenic	8p23.1d	T	0.0822	92.64	4.31 × 10^−5^
rs11586310	*IRF2BP2*	intergenic	1q42.3a	G	0.0625	−104.3	4.38 × 10^−5^
rs2519580	*TFPI2*	intergenic	7q21.3a	T	0.1466	−71.32	4.62 × 10^−5^
rs2519573	*TFPI2*	intergenic	7q21.3a	T	0.1469	−71.19	4.69 × 10^−5^
rs2724079	*TFPI2*	intergenic	7q21.3a	A	0.1475	−70.25	5.77 × 10^−5^
rs11783707	*MFHAS1,CLDN23*	intergenic	8p23.1d	T	0.0627	103.9	6.10 × 10^−5^
rs2093145	*CST9*	intergenic	20p11.21b	A	0.2963	−54.71	6.10 × 10^−5^
rs1888286	*ASTN2*	intronic	9q33.1b	G	0.3201	53.33	6.51 × 10^−5^
rs11587639	*IRF2BP2*	intergenic	1q42.3a	C	0.0608	−103.2	6.72 × 10^−5^
rs11780486	*MFHAS1*, *CLDN23*	intergenic	8p23.1d	C	0.0625	103.4	6.74 × 10^−5^
rs337999	*GALNT17*	intronic	4q34.1b	G	0.2463	57.03	6.83 × 10^−5^
rs2987460	*IRF2BP2*	intergenic	1q42.3a	T	0.0727	−94.17	7.74 × 10^−5^
rs337997	*GALNT17*	intronic	4q34.1b	T	0.2458	56.61	7.82 × 10^−5^
rs853334	*FGD5*, *C3ORF20*	intergenic	3p24.3e	A	0.4373	−49.31	7.92 × 10^−5^
rs11111767	*NT5DC3*	intronic	12q23.3a	A	0.3923	49.77	8.12 × 10^−5^
rs7083122	*RHOBTB1*	intronic	10q21.2a	A	0.1486	68.83	8.67 × 10^−5^
rs1928980	*ASTN2*	intronic	9q33.1b	A	0.3152	52.4	8.76 × 10^−5^
rs2421930	*DDX18*	intergenic	2q14.1d	G	0.0290	147	8.77 × 10^−5^
rs1928984	*ASTN2*	intronic	9q33.1b	C	0.3157	52.33	8.81 × 10^−5^
rs3751204	*NT5DC3*	utr-variant-3-prime	12q23.3a	T	0.3783	49.85	8.86 × 10^−5^
rs10124001	*JAK2*, *RCL1*, *MIR101-2*	intergenic	9p24.1c	A	0.1146	−76.5	9.19 × 10^−5^
rs1265074	*CCHCR1*	intronic	6p21.33a	A	0.3225	−51.96	9.23 × 10^−5^
rs2493869	*CDKAL1*	intronic	6p22.3b	A	0.3373	−51.44	9.39 × 10^−5^
rs10495350	*IRF2BP2*	intergenic	1q42.3a	T	0.0613	−100.4	9.45 × 10^−5^
rs2446484	*CDKAL1*	intronic	6p22.3b	G	0.3213	−51.99	9.66 × 10^−5^
rs10989864		intergenic	9q31.1b	A	0.0428	117.6	9.94 × 10^−5^
rs4344422	*ADRA2A*	intergenic	10q25.2b	G	0.0910	83.98	1.00 × 10^−4^

^a^ MAF stands for Minor allele frequency; ^b^ Coefficient from single-marker association test with age, sex, area, and body mass index included as covariates; ^c^ *p*-values from single-marker association test indicates *p*-value = 10^−5^. Although these SNPs did not achieve the genome-wide level significance, 41 genetic variants (listed in Table 1) emerged among the 344,893 SNPs to have some evidence of association with PA under *p*-value < 10^−4^, and they were mapped to 27 genes.

#### 2.1.3. Multiple SNP-Based Association Analysis

The multi-stage procedure was applied to identify multiple causal SNPs. After performing single-SNP association tests in the first stage, we chose top 1000, top 2000, top 3000, and top 4000 SNPs that exhibit the strongest individual associations with PA. During the next stage, 639, 1248, 1760, and 2239 from the respective top SNP groups were jointly identified as PA-related genetic variants by the EN regularization method. The elastic-net allows correlation among predictors in variable selection, so there can be different selection results according to pre-screened datasets [11]. At the validation stage, these jointly identified SNPs were further evaluated using the bootstrap selection stability (BSS) measure. Since the lists of SNPs were different depending on the number of pre-screened SNPs, we focused on the common 457 SNPs that were simultaneously identified from all four groups. These commonly selected variants have higher BSS than the variants which are chosen only one pre-defined dataset [11]. Table 2 lists the final 59 variants selected with BSS ≥0.95 and mapped to 76 known genes. The *p*-values were calculated using a multiple linear regression model with adjustment for sex, age, area, and body mass index. Finally, pathway analysis found one pathway enriched in these 76 genes: Maturity onset diabetes of the young (MODY). This pathway includes *GCK* and *HES1* genes and has a *p*-value of 0.076.

**Table 2:** SNPs associated with physical activity with bootstrap selection stability (BSS) ≥95% in top 1000, top 2000, top 3000, and top 4000 SNPs through a multi-stage approach.

rs Number	Gene Symbol	Location of SNP	Cytoband	Minor Allele		MAF ^a^		Effect Size (4000) ^b^	BSS (4000) ^c^	*p*-Value ^d^ (4000)
rs10849033	CCND2, C12ORF5	intronic	12p13.32a	C		0.4886		19.799	99.7	0.00003
rs4252821	CCNI	Downstream (500 bp) Upstream (5000 bp)	4q21.1b	G		0.1013		15.281	96.9	0.00003
rs853334	FGD5, C3ORF20	intronic	3p24.3e	A		0.4373		−15.166	98.3	0.00009
rs17099857	ARHGAP26	intergenic	5q31.3e	C		0.0763		16.107	99.2	0.00010
rs4906747	ATP10A	intergenic	15q12a	G		0.0640		14.613	97.4	0.00010
rs6030844	RNU6-1, RNU6-2	intergenic	20q13.11b	C		0.1729		14.352	97.6	0.00010
rs10978130	PTPRD	intergenic	9p23d	C		0.1523		23.022	99.9	0.00013
rs10507652	TDRD3	intergenic	13q21.2b	T		0.0536		−19.779	99.9	0.00015
rs7649230	HES1	intergenic	3q29c	C		0.3382		12.115	96.4	0.00017
rs13106655	TMEM156	nonsynonymous	4p14c	G		0.2674		13.811	98.2	0.00018
rs16953182	UNC13C	intronic	15q21.3b	G		0.0165		17.941	99.2	0.00021
rs7976955	VWF, TMEM16B	utr-variant-3-prime	12p13.31e	T		0.0230		12.674	95.5	0.00025
rs2586038	MRPS23	intergenic	17q22d	G		0.3314		−14.089	97.1	0.00026
rs9833833	UBE2E1	intergenic	3p24.3a	T		0.3393		16.227	99.3	0.00031
rs41455146	ADAM12	intergenic	10q26.2a	G		0.0726		−12.430	96.5	0.00033
rs2314612	GPR149, MME	intronic	3q25.2c	A		0.4665		−20.284	99.6	0.00033
rs10513868	DLGAP1, FLJ35776	intronic	18p11.31e	G		0.2335		13.314	97.6	0.00035
rs4131468	MBD2, DCC, SNORA30, SNORA37	intergenic	18q21.2c	T		0.4954		−15.354	98.8	0.00036
rs2851651		intergenic	11q22.1a	T		0.2047		−15.510	99	0.00039
rs2728504	ZNF521	intergenic	18q11.2d	T		0.2713		−19.259	96.9	0.00042
rs17339892	MCTP1	intergenic	5q15c	T		0.1076		12.451	96.6	0.00051
rs7997236	FAM155A	intergenic	13q33.3a	A		0.0498		−20.321	99.7	0.00054
rs1387243	FAR2, RN5S1, CCDC91	intergenic	12p11.22b	C		0.1766		12.015	98.4	0.00056
rs707586	AJAP1	intergenic	1p36.31b	G		0.2672		−18.571	99.7	0.00064
rs4978521	ZFP37, SLC46A2	intergenic	9q32b	T		0.0886		−19.155	96.7	0.00066
rs2067730	NRXN3	utr-variant-3-prime	14q31.1a	C		0.0308		−11.340	96.2	0.00072
rs16967978	LOC100132540, LOC339047, XYLT1	intronic	16p12.3c	A		0.0427		13.550	95.2	0.00073
rs41351947	EIF2B3	intergenic	1p34.1d	C		0.0291		−15.446	99.2	0.00073
rs931701	BOC	intronic	3q13.2b	A		0.3798		−15.920	98.9	0.00077
rs729239	RNU6-1, RNU6-2	intronic	4q21.1b	T		0.0194		−15.186	98.4	0.00080
rs10020466	RN5S1	intronic	4q34.3d	C		0.0739		−13.149	98.4	0.00082
rs1536053	C13ORF16	intronic	13q34b	T		0.0393		−15.016	96.8	0.00083
rs17553316	RGNEF	intergenic	5q13.2c	G		0.0192		12.629	96.7	0.00094
rs445942	C7ORF10, INHBA	intronic	7p14.1b	C		0.1666		−16.716	99	0.00099
rs17058450	FAM116A	intergenic	3p14.3a	T		0.0742		−12.336	96.1	0.00103
rs11167061	FLJ43860	Upstream (5000 bp)	8q24.3d	A		0.2238		−15.508	99.2	0.00112
rs1453282		intronic	7p12.3b	C		0.3057		−16.260	99.5	0.00130
rs4864029	RNU6-1, RNU6-2	intergenic	4q28.3b	G		0.1181		17.370	99.4	0.00134
rs4620043	LIFR	intergenic	5p13.1c	A		0.2291		11.716	95.3	0.00153
rs2140340	CSMD1	intronic	8p23.2c	T		0.0826		15.130	98.4	0.00177
rs3738178	MOSC1	intergenic	1q41d	A		0.0966		13.128	96.3	0.00189
rs7770227		intergenic	6q22.1b	T		0.0781		18.199	99.7	0.00192
rs17730347	MCTP2	intronic	15q26.2a	C		0.2599		13.531	96.4	0.00194
rs11024787	PTPN5	intronic	11p15.1c	A		0.0300		−18.894	99.9	0.00200
rs1605987	EDIL3	intergenic	5q14.3b	T		0.1921		−14.930	98.2	0.00204
rs3802292	CSMD1	intronic	8p23.2d	T		0.3660		−15.587	99.8	0.00238
rs2273635	KIAA1305	intronic	14q12a	T		0.0956		13.632	97.8	0.00243
rs7102454	CFL1, OVOL1, SNX32	intronic	11q13.1d	C		0.3163		−13.331	96.8	0.00299
rs2725795	C15ORF53	intergenic	15q14d	G		0.0710		17.092	99.2	0.00323
rs2280732	PLB1	intergenic	2p23.2b	C		0.2716		11.855	97.2	0.00324
rs3025365	DBH, FAM163B	intergenic	9q34.2a	C		0.1761		11.904	95.1	0.00326
rs6979515	NXPH1	intergenic	7p21.3d	G		0.3828		−18.242	99.2	0.00364
rs12332121	RPS17P2	intronic	5q23.1a	C		0.1237		−15.236	98.4	0.00445
rs10046269	EYA4, TCF21	intergenic	6q23.2c	C		0.0454		17.753	99.4	0.00484
rs4921144	MIR146A, ATP10B	Upstream (5000 bp)	5q33.3d	A		0.0454		−12.473	96.4	0.00495
rs888053	VIT, STRN	intronic	2p22.2b	A		0.2656		14.279	96.5	0.00512
rs1079082	ZNF579, FIZ1	intronic	19q13.42c	T		0.1132		13.545	96.4	0.00589
rs4531650	EGLN3, C14ORF147	intronic	14q13.1c	C		0.3818		−14.878	98.3	0.00643
rs1799884	GCK, YKT6	intergenic	7p13d	A		0.1892		−11.829	96.7	0.00724

^a^ MAF stands for Minor allele frequency; ^b^ Effect size obtained from top 4000 SNPs; ^c^ BSS in top 4000 SNPs; ^d^ *p*-values from multiple regression.

**Figure 3:**
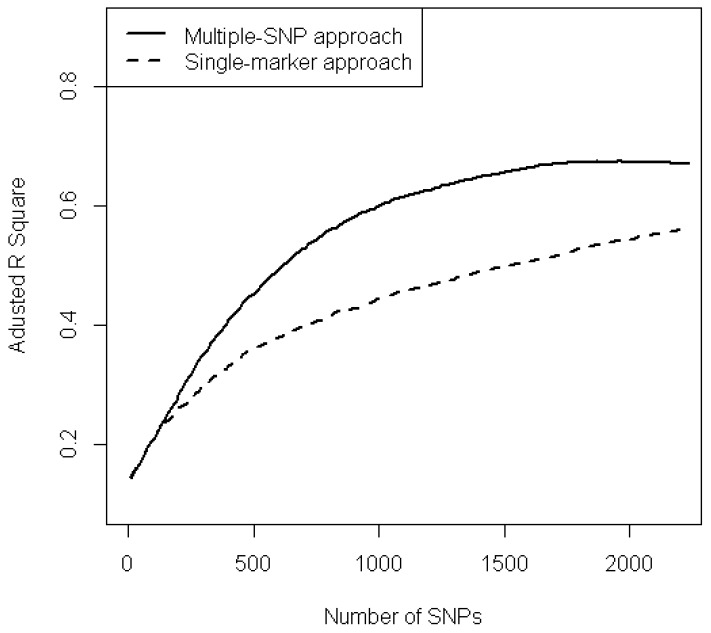
Phenotype variation between multi-stage approach (solid line) and single-marker approach (dashed line).

The predictive power of the 2239 SNPs identified from the top 4000 SNPs was next investigated. SNPs were ranked in order form smallest to largest and selected using a given BSS cut-off value, 0.95. Then, a multiple regression model with the selected SNPs was fitted to compute the corresponding adjusted *R*^2^ value. Figure 3 compares the adjusted *R*^2^ values *versus* number of SNPs between the multiple SNP analysis and the single-marker approach. It is clear that the predictive power increases with the number SNP for both approaches, but the multi-stage approach always performs better than the single-marker approach for prediction purpose. This shows that multi-stage approach using a BSS cut-off value provides a better explanation of phenotype than the single marker approach.

### 2.2. Discussion

The present study investigated genetic factors associated with PA for the Korean population, by performing large-scale GWA through single-SNP analysis and multiple SNP analysis via the EN regularization method. Single-SNP association tests are appropriate to determine individual associations between each SNP and the trait or phenotype. However, if the purpose is to predict the phenotype, then the joint identification of genetic factors would be powerful and provide a better prediction of the trait when multiple genetic factors exist for a common complex trait. In the presence of multicollinearity due to linkage disequilibrium among SNPs, EN regularization with BSS offers more accurate identification of multiple SNPs than ordinary multiple regression analysis.

Our single-marker analysis results showed that, although the most significant SNP did not attain the genome-wide significance level (rs7023003, *p*-value = 4.67 × 10^−6^), 41 SNPs did exhibit some evidence of association with PA at a less stringent significance level (*p*-value < 10^−4^). Among the 27 genes identified, *CDKAL1*, CDK5 regulatory subunit associated protein 1-like 1, is one of the tailfloated protein family and associated with a type 2 diabetes susceptibility gene responsible for tRNALys modification [16]. *CDKAL1* explores the TCR40/Get3 assisted pathway for insertion of its *C*-terminal transmembrane domain into the endoplasmic reticulum [16]. It has been reported that this gene can influence insulin response and risk of type 2 diabetes [17].

*TFPI2*, tissue factor pathway inhibitor 2, is found to be a tumor suppressor gene and frequently inactivated through promoter methylation in several kinds of tumors [18]. In particular *TFPI2* methylation plays a key role in the diagnosis of colorectal cancer and has been demonstrated to exist in colorectal cancer patients’ sera [19].

*CCHCR1*, also called coiled-coil alpha-helical rod protein 1, is a candidate gene for Psoriasis which is one kinds of chronic inflammatory skin disorder [20]. *CCHCR1* gene is found to be expressed in psoriatic lesions compared to normal healthy skin or other hyper proliferative skin disorders [21,22]. *CCHCR1* has been demonstrated to be involved in steroidogenesis of the skin [20].

Another gene, *RHOBTB1*, encodes Rho-related BTB domain-containing protein 1 [23]. The protein encoded by this gene belongs to the Rho family of the small GTPase superfamily. It has a GTPase domain, a proline-rich region, a tandem of 2 BTB (broad complex, tramtrack, and bric-a-brac) domains, and a conserved *C*-terminal region. The protein plays a role in small GTPase-mediated signal transduction and the organization of the actin filament system. Alternate transcriptional splice variants have been characterized. It is known that the gene is highly expressed in skeletal muscle, stomach, placenta, kidney, and testis.

The single-marker analysis also identified *ASTN2*. It encodes a protein that is expressed in the brain and may function in neuronal migration, based on functional studies of the related *astrotactin 1* gene in human and mouse. A deletion at this locus was shown to be associated with schizophrenia [24]. Multiple transcript variants encoding different proteins have been identified in this locus.

Through the multi-stage approach, subsets of multiple SNPs were jointly identified through the EN regularization method. Among the 457 common SNPs found, 59 SNPs with BSS values exceeding 0.95 were mapped to 76 genes. Of these genes, *ADAM12* encodes ADAM metallopeptidase domain 12 and is a disintegrin and metalloproteases family member [25]. *ADAM12* is also a multidomain type I transmembrane protein that functions both in normal physiology and in diseases [25]. *CCNI* gene, cyclin I, is also identified. *CCNI* is known to be expressed in human forebrain cortex [26]. *CCNI* is also presented in skeletal muscle, heart, and brain and expressed constantly during cell cycle progression [26]. Moreover, *PTPN5* encodes protein tyrosine phosphatase, non-receptor type 5 and involves in regulating the occurrence of abnormal stress responses underlying depression-related disorders [27]. The basal levels of *PTPN5* expression in the dorsal hippocampus determine an individual’s susceptibility for developing stress-related cognitive and morphological changes [27]. Another gene found, *NRXN3* (neurexin 3), is a member of the neuroxines protein family that acts in the vertebrate nervous system as cell adhesion molecules and receptors [28]. The mutations of *NRXN3* are related to alcohol and nicotine dependence in patients who suffer from schizophrenia [29,30].

Pathway analysis of the 59 SNPs led to the identification of MODY. Its inherence is responsible for non-insulin-dependent diabetes typically diagnosed among young people, especially under 25 years of age [31,32]. MODY is often referred to as monogenic diabetes, which is thought to be different from type 1 and type 2 diabetes [33,34]. MODY is also known to include the *GCK* gene and the *HES1* gene. The gene *GCK*, glucokinase, is one of the frequently causing MODY genes and accounts for approximately 35% of cases [33]. *HES1*, Hairy enhancer of split 1, is one of the highly conversed family of Hairy related basic helix-loop-helix (bHLH) proteins [35]. *HES1* is also known to be involved specifically in Notch1 signaling in neural cells and in bone narrow [35].

The main limitation of our study concerns the self-reporting nature of PA and the questions used were not sufficiently detailed. In addition to possible recall error by the participants, the phenotypes of total PA level may not be well defined. PA was classified by five categories based on MET intensities with average MET assigned to each category. The estimated total PA level may dilute MET intensities and lead to a low power for the genetic association study. Furthermore, it is not feasible to compare our results with previous studies in the absence of similar genotyped SNPs. Consequently, we adopted the bootstrap sampling scheme to obtain replication data sets with appropriate selection stability measure to confirm the findings [11].

## 3. Experimental Section

### 3.1. Subjects

Study subjects were selected from an ongoing population-based cohort, as part of the Korean Genome and Epidemiology Study (KoGES). Participants were recruited from residents in two cities (Ansung and Ansan) in Gyeonggi-do, Korea. We enrolled 10,038 males and females between 2001–2002 for a baseline study, whose demographics have been reported [8]. The Korean Genome and Epidemiology Study was launched in 2007, whereby over 10,000 subjects were recruited from two community-based cohorts: the rural Ansung and urban Ansan cohorts in the Gyeonggi of Korea. The initial samples included 5018 and 5020 participants aged 40 to 69 years from the two cohorts, respectively [36]. Table 3 summarizes the demographic characteristics of the participants. There were more female participants in Ansung than Ansan but the Ansung cohort was on average older than the Ansan cohort, reflecting the differences between rural and urban areas. This study obtained approval from the appropriate institutional review boards of each participating institution, and written informed consent was obtained from all participants.

**Table 3:** Demographic characteristics of participants in the Korean cohorts.

Cohort	Sex (*n*)			Age (Mean ± SD)		
	Male	Female	Both	Male	Female	Both
**Ansung (rural)**	1658	2240	3898	55.92 ± 8.66	55.65 ± 8.81	55.77 ± 8.75
**Ansan (urban)**	2337	2219	4556	48.56 ± 7.44	49.60 ± 8.22	49.07 ± 7.85
**Total**	3995	4459	8454	51.61 ± 7.44	52.64 ± 9.04	52.16 ± 8.92

### 3.2. Physical Activity Information

Information on intensity and duration of daily PA was obtained from each participant using a structured questionnaire that included five components on PA: stable (lying down except sleeping), sitting (e.g., during typing, playing cards, driving, office work, attending a class), low intensity (e.g., walking, doing the laundry, cleaning, leisure time ping pong), medium intensity (e.g., walking, carpentering, regular exercise, badminton, swimming, tennis), high intensity (e.g., sports competition, climbing, running, logging, farming). For each type of PA, its duration was measured in minutes. Since each question contained multiple PAs with varying MET intensities, the average MET intensity was assigned. The total amount of daily PA was then calculated by summing across the products of the average MET and the corresponding durations [37].

### 3.3. Genotypes

Genomic DNA samples were isolated from peripheral blood drawn from the participants. The majority of genomic DNAs were genotyped on the Affymetrix Genome-Wide Human SNP array 5.0 containing 500,568 SNPs. From the total of 10,038 participants, 10,004 available samples were genotyped [36] by implementing Bayesian robust linear modeling with the Mahalanobis distance (BRLMM) algorithm, and standard quality control procedures were adopted. Samples with high missing genotype call rate (>4%, *n* = 401), high heterozygosity (>30%, *n* = 11), gender inconsistencies (*n* = 41), and those obtained from individuals who had developed any form of cancer (*n* = 101), were excluded from subsequent analyses, along with related or identical individuals whose computed average pairwise identity-by-state value was higher than that estimated from first-degree relatives of Korean sib-pair samples (>0.80, *n* = 601). Samples whose genotype-inferred sex disagreed with clinical records were re-tested for sex confirmation using the SNaPshot Multiplex System (Applied Biosystems, Life Technologies, Carlsbad, CA, USA). Markers with high missing gene call rate (>5%), low Minor Allele Frequency (MAF) (<0.01) and significant deviation from Hardy-Weinberg equilibrium (*p*-value < 1 × 10^−6^) were excluded, leaving a total of 352,228 markers to be examined among 8842 individuals. For multiple SNP analysis, reduced information was common due to missing values. To get complete genotype data, we imputed missing genotypes using the Fastphase software (University of Washington, Seattle, WA, USA) [38]. As a result, a total of 344,893 SNP markers with chromosomes 1–22 were obtained for our study.

### 3.4. Statistical Analysis

Single-SNP association analysis was first performed for each of the 344,893 SNPs using linear regression analysis with adjustments for sex, age, area, and body mass index. A multi-stage approach [11] was next conducted to identify multiple SNPs associated with PA. At the first stage, through single-SNP analysis, we selected the top 1000, top 2000, top 3000, and top 4000 SNPs which exhibit strong associations with PA for dimensional reduction. At the second stage, multiple-SNP analysis was performed with the EN regularization method, by utilizing a subset of SNPs chosen at the first stage. At the final stage, the bootstrap selection stability (BSS) measure was computed for each SNP, which indicated how consistently a SNP was replicated in bootstrap datasets. SNPs with high BSS values tend to have a higher chance of being replicated. More information about a multi-stage approach is described in Cho *et al.* [11].

To investigate the biological significance of PA-related genetic variants, we mapped the identified SNPs to an exon/intron and performed pathway enrichment analysis. All pathways related to the identified genes were investigated via the Kyoto Encyclopedia of Genes and Genomes (KEGG) database. Pathways were evaluated by the over-representation statistic and the Expression Analysis Systematic Explorer (EASE, Database for Annotation, Visualization and Integrated Discovery (DAVID), Frederick, MD, USA) score [39]. EASE calculates over–representation using Fisher’s exact probability with respect to the total number of genes assayed and annotated within each system to measure the gene-enrichment in annotation terms [39]. *p*-values less than 0.1 defined as significantly regulated pathway. The single-marker association tests and adopted multi-stage approach were conducted using the PLINK [40] and R [41] software.

## 4. Conclusions

In this study, we demonstrated that the joint identification of SNPs could enable the identification of multiple SNPs with good predictive power for PA and a pathway enriched for PA. Previous genetic studies have focused on the relationship between PA and health (or fitness) [37,42,43,44]. Future studies are recommended to determine pertinent genetic factors that influence health- or fitness-related PA. In view of the large population diversities in GWA studies, a systematic comparison is needed between our Korean results and those derived from other populations.
